# Names with /i/ Suit Positive Faces: The Naming Paradigm

**DOI:** 10.5334/joc.466

**Published:** 2025-10-27

**Authors:** Anita Körner, Larissa Röth, Ralf Rummer

**Affiliations:** 1 Department of Psychology, University of Kassel, Holländische Str. 36–38, 34127 Kassel, Germany

**Keywords:** sound symbolism, iconicity, valence, names, naming paradigm

## Abstract

Features of word form (e.g., the vowel *i* as in *meet*) are associated with word meaning (e.g., positive valence), termed sound symbolism. Experimentally, sound symbolism is predominantly examined using pseudo-words. The present research employs a new experimental paradigm where participants are shown faces and are asked to choose a suitable name from memory for each face. In two experiments (total *N* = 399), we tested whether valence (manipulated via facial expressions, Experiment 1a, or likability, Experiment 1b) influences the occurrence of i-phonemes and o-phonemes in first names. To test convergent validity, a corpus analysis (Study 2) examined the association of likability and the occurrence of i-phonemes and o-phonemes using a representative corpus of German first names. Consistent with previous findings, names given to positively (vs. negatively) valenced faces more frequently contained i-phonemes, whereas, unexpectedly, valence did not influence o-phoneme occurrence. Thus, the naming paradigm bridges the gap between controlled pseudo-word experiments and the natural use of real names and can be employed to examine whether sound symbolic associations are stable enough to generalize to meaningful words.

First names and faces frequently seem to fit. For example, more conservative looking persons frequently seem to have traditional names. Participants agree above chance level on which names suit given faces (Lea et al., 2007), and these names are disproportionately the correct ones (Zwebner et al., 2017; 2024). Moreover, both faces and names have been found to influence person evaluations (Nett et al., 2020; Zebrowitz & Montepare, 2008). The present research examines associations between names and faces, narrowing down on sublexical name features. Specifically, we examine whether vowels in first names are associated with face valence. For this, we propose a new experimental paradigm, where participants are asked to choose a first name from memory that fits a depicted face.

## Valence Sound Symbolism

Aspects of word form can carry cues about word meaning, a phenomenon termed sound symbolism or iconicity (Perniss et al., 2010; Schmidtke et al., 2014; Sidhu & Pexman, 2018; Winter et al., 2017). A prominent example concerns shape: When asked to match pseudo-words to shapes, most participants match *Bouba* with round shapes and *Kiki* with spikey shapes (Ćwiek et al., 2022; Köhler, 1929). In addition to shape, sound symbolic associations have been found for other dimensions, such as color (Cuskley et al., 2019; Simner et al., 2005), size (Sapir, 1929; Thompson & Estes, 2011), and abstraction (Maglio et al., 2014). Sound symbolic associations facilitate cognitive processing, so that congruent stimulus–pseudo-word combinations facilitate learning (Lockwood et al., 2016; Nygaard et al., 2009; Sonier et al., 2020) and speeded responses (Parise & Spence, 2012; Schmidtke et al., 2024; Vainio 2021), indicating that sound symbolism is deeply engrained in cognitive processing.

The present research examines valence sound symbolism—sound symbolic associations with emotional valence. Among consonants, nasals (e.g., /n/), especially at the beginning of words, have been found to be associated with negative valence in Indo-European languages (Auracher et al., 2010; de Zubicaray et al., 2023; de Zubicaray & Hinojosa, 2024; Louwerse & Qu, 2017) and with positive valence in Chinese (Louwerse & Qu, 2017). Plosives (e.g., /p/) have been mostly found to be associated with negative valence (see e.g., Aryani et al., 2018; Benczes & Kovács, 2022; Miall, 2001; Motoki et al., 2022; Whissell, 1999; de Zubicaraya et al., 2023; however, see Auracher et al., 2010; Wiseman & van Peer, 2003). And consonants articulated at the front (vs. back; e.g., /b/ vs. /g/) of the mouth are associated with positive (vs. negative) valence (Folkins & Lenrow, 1966; Maschmann et al., 2020; see also Benczes & Kovács, 2022; Miron, 1961; Myers-Schulz et al., 2013).

In the present research, we concentrate on associations between valence and vowels. Specifically, we examine the phenomenon that i-phonemes (/i/ as in English *meet or* /ɪ/ as in English *hit*) are associated with positive valence (e.g., with smiling faces and likable people) while rounded vowels (e.g., /o/ and /u/ as in French *rose* and English *food*) are associated with negative valence (e.g., with sad faces and less likable people; Karthikeyan et al., 2016; Körner & Rummer, 2022; Rummer & Schweppe, 2019; Schmidtke et al., 2024; see also Crockett, 1970; de Zubicaray & Hinojosa, 2024; Miron, 1961; Lacey et al., 2024; Tarte, 1982; Whissell, 2000). This vowel–valence association has been observed for languages from several language families, specifically Indo-European (Rummer & Schweppe, 2019), Sino-Tibetan (Yu et al., 2021), Japonic (Körner & Rummer, 2023), and Uralic (Benczes & Kovács, 2022). Moreover, it has been found in corpus analyses (Yu et al., 2021; Benczes & Kovács, 2022), when participants invented pseudo-words or pseudo-names (Körner & Rummer, 2023; Rummer & Schweppe, 2019), and when participants selected or rated pseudo-words (Garrido & Godinho, 2021; Körner & Rummer, 2022).

Vowel–valence associations have been shown to be driven by overlapping muscle tension for articulation and facial expressions. Specifically, articulating /i/ and smiling requires tension in lip spreading muscles (e.g., the zygomaticus major muscle), while articulating rounded vowels requires tension in antagonistic muscles (e.g., the orbicularis oris muscle). The overlap in muscle tension could have led to valence that is associated with facial expressions getting also associated with vowels (Körner & Rummer, 2022; Rummer et al., 2014). Thus, the theoretical basis for vowel–valence associations, at least with respect to /i/ compared to rounded vowels, lies in an embodied mechanism involving a valence transfer from facial expression to vowels via overlapping muscle tension for facial expressions and articulation.

## Empirical Paradigms

Although several empirical paradigms exist (e.g., Punselie et al., 2024), sound symbolism is typically examined with either pseudo-word experiments or analyses of language corpora (Nielsen & Dingemanse, 2021). Using pseudo-words has the advantage of maximal experimental control but the disadvantage of poor ecological validity. Thus, it is unclear whether observed associations generalize from nonsense-stimuli to real words. Similarly, ecological validity is also low for paradigms where participants invent pseudo-words (e.g., Berlin, 2006; Rummer et al., 2014; Saji et al., 2019) or pseudo-names (Körner & Rummer, 2023; Rummer & Schweppe, 2019).

In corpus analyses, in contrast, natural language is examined for statistical associations. This has the advantage of high ecological validity but it is unclear whether associations are only statistical instead of psychological (e.g., some consonant features are more frequent in verbs than other word classes, such as coronals in Japanese and bilabials in French, which might not reflect a psychological association; Dingemanse et al., 2015). Moreover, language changes might have watered down associations between word form and meaning. For example, vowel shifts might have changed words so that vowels no longer concur with word meaning.^1^ Thus, neither finding a statistical association nor finding no statistical association in corpus analyses is conclusive about whether there is a psychological association.^2^ Combining the advantages of both experimental control and ecological validity, we propose a new experimental paradigm—the naming paradigm—where participants retrieve names from memory that they consider suitable for a given stimulus.

The experimental examination of real first names has high ecological validity. In contrast to pseudo-words, first names carry social meaning. People rely on first names to infer persons’ socio-demographic background (Barlow & Lahey, 2018), such as age (Rudolph et al., 2007), class and race (Gaddis, 2017), as well as various other personal and social attributes, such as attractiveness (Garwood et al., 1980) and personality traits (Mehrabian, 1997; Moss-Racucin et al., 2012; Nett et al., 2020). First names have even been found to predict important life outcomes, such as academic performance (Erwin & Calev, 1984; Harari & McDavid, 1973), social success (Busse & Seeaydarian, 1979; Gebauer et al., 2012; Greitemeyer & Kunz, 2013; McDavid & Harari, 1966), and chances on the job market (Bertrand & Mullainathan, 2004). Thus, names are highly ecologically relevant (Sidhu & Pexman, 2019).

Previous research on sound symbolism in first names typically required participants to choose between two experimenter-provided names (e.g., Joanna vs. Erica; Barton & Halberstadt 2018; Sidhu & Pexman, 2015; Sidhu et al., 2019). In contrast, the present research employs an unrestricted paradigm in which participants could select any first name that seems fitting for a given face. This has the advantage of not introducing confounds (in contrast to name pairs). Moreover, whereas name pairs have to be pre-selected to vary only on the target dimensions, participant-provided names can be examined for any type of linguistic feature; thus, the resulting data are rich and enable multitudinous analyses. Participant-provided names can reflect complex interactions of linguistic features within words, enabling advanced statistical models that take, for example, complex syllable structures into account. In sum, (a) proper names are a highly suitable type of linguistic stimuli for examining sound symbolism and (b) using an unrestricted paradigm where participants can select any name is a superior method of using proper names in sound symbolism research.

The present research employs the naming paradigm to examine valence sound symbolism, testing whether valence–vowel associations are so robust as to persist when participants are asked to provide real names for valenced stimuli. We hypothesized to replicate previous findings (e.g., Körner & Rummer, 2023; Rummer & Schweppe, 2019), specifically we hypothesize that i-phonemes are associated with positive valence and o-phonemes are associated with negative valence.

## Experiments 1a–b: Giving Names to Positive and Negative Faces

### Method

Valence was operationalized using manipulations employed in previous experiments on sound symbolism, via facial expressions (happiness vs. anger; modeled after Körner & Rummer, 2023) in Experiment 1a and via face likability (modeled after Körner & Rummer, 2022; Experiments 3–4) in Experiment 1b. Both manipulations have been shown to generalize across cultures. Specifically, happy and angry facial expressions show cross-cultural similarities (e.g., Brooks et al., 2024; Elfenbein & Ambady, 2002) even concerning valence sound symbolism (Körner & Rummer, 2023), and there is evidence that likability/warmth is one of the major person perception dimensions across many cultures (Jones et al., 2021; Thalmeyer et al., 2025).

#### Participants

German-speaking participants were recruited through SurveyCircle (https://www.surveycircle.com) in exchange for mutual study participation and a charitable donation on their behalf or through a local participant pool in exchange for partial course credit (only Experiment 1b). In Experiments 1a and 1b, 162 and 261 participants completed the experiment, respectively. According with the pre-registrations (https://osf.io/478ay/ and https://osf.io/j36sk/), participants who used words instead of first names more than five times (Experiment 1a: *N* = 1; Experiment 1b: *N* = 1), who used the same first name more than three times (Experiment 1a: *N* = 4; Experiment 1b: *N* = 11), and who were advanced local psychology students (Experiment 1a: *N* = 1; Experiment 1b: *N* = 6) were excluded from all analyses. In Experiment 1a, the final sample consisted of 156 participants (105 female, 51 male; *M*_age_ = 28, *SD*_age_ = 9), yielding a power of 80% (with a = .05) for finding *d*_z_ = 0.20 in the one-tailed pairwise comparisons; and in Experiment 1b, the final sample consisted of 243 participants (169 female, 74 male; *M*_age_ = 25, *SD*_age_ = 7), yielding a power of 80% (with a = .05) for finding *d*_z_ = 0.16 in the one-tailed pairwise comparisons.

#### Material

**Figure 1:**
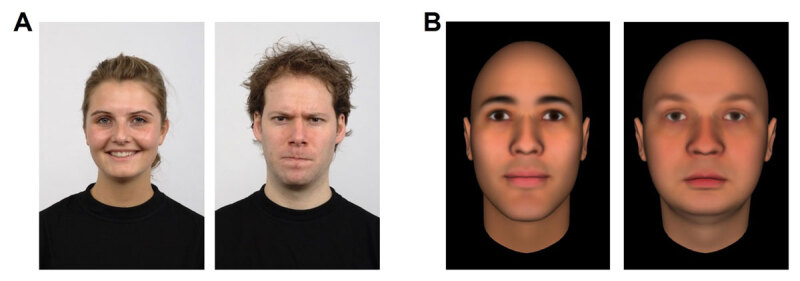
Examples of Stimuli Used in Experiment 1a (Panel A) and Experiment 1b (Panel B). *Note*. Participants were asked to select any first name from memory that, in their opinion, suited the depicted face. The face on the left side of each panel is a positive one (A: positive emotional expression; B: high in likability) and the face on the right side is a negative one (A: negative emotional expression; B: low in likability). For more information, see Langner et al. (2010) and Todorov et al. (2013); faces were created using the FaceGen software, http://facegen.com, to differ in likability).

In Experiment 1a, valence was manipulated via facial expression. For this, 30 individuals from the Radboud Faces Database (Langner et al., 2010) were selected, 16 females and 14 males. From each, one picture with a positive emotional expression (happiness) and one with a negative emotional expression (anger) were selected. Two parallel picture sets were created, so that each model was present in each set (either with a positive or with a negative expression) and each set contained fifteen positive and fifteen negative pictures (see Figure 1, Panel A; sets were balanced across participants).

In Experiment 1b, face valence was manipulated via likability. For this 30 male faces, 15 positive and 15 negative (nine White, three Black, and three East Asian, each) faces from (Todorov et al. 2013; Oosterhof & Todorov, 2008; Todorov & Oosterhof, 2011; Todorov & Oh, 2021) were selected. For positive (negative) valence, faces that were 2 or 3 standard deviations above (below) the baseline in likability were selected (see Figure 1, Panel B).

#### Procedure

After providing informed consent, participants were presented with one face per trial and were asked to select a fitting first name from memory. They were instructed to choose only existing first names, that is, names they had already heard or read as first names, and to not repeat any previously used names. Each trial consisted of a face and a prompt to type in a name. After providing names for all 30 faces (in random order and self-paced), participants answered demographic questions, were asked to speculate about the aim of the experiment, and could leave a comment.

#### Analysis

Existing words in German or English were excluded from analyses (Experiment 1a: 4 trials; Experiment 1b: 2 trials). The phonemes in each name were coded by listening to the German pronunciation from a website of first names (https://www.baby-vornamen.de). Coders used the International Phonetic Alphabet (International Phonetic Association, 1999) to mark occurrences of i-phonemes (including /i:/, /i/, and /ɪ/) and o-phonemes (including /o:/, /o/, and /ɔ/). The occurrences of i-phonemes and o-phonemes were counted; for example, in the name Dominik, the phonetic coding resulted in D/ɔ/m/i/n/ɪ/k, yielding i-phonemes = 2 and o-phonemes = 1. The number of i-phonemes and o-phonemes were then averaged across trials, separately for positive and negative valence. These means were entered into a 2 (valence: positive vs. negative) 2 (vowel: i vs. o) repeated measures ANOVA. Additionally, we compared the influence of valence in i-phoneme occurrence and o-phoneme occurrence in within-participant *t*-tests. As pre-registered, all *t*-tests were one-tailed.

#### Transparency and Openness

We report how we determined our sample size, all data exclusions, all manipulations, and all measures. All data, analysis scripts, materials, and links to the pre-registrations (including design, hypotheses, and analysis plan) can be found at https://osf.io/925ky/. We deviate from the pre-registration by reporting simple effects even when interactions fail to reach significance and by reporting two-tailed instead of one-tailed *t*-tests. Data were analyzed using R (version 4.2.1, R Core Team, 2022).

### Results

#### Experiment 1a

**Figure 2:**
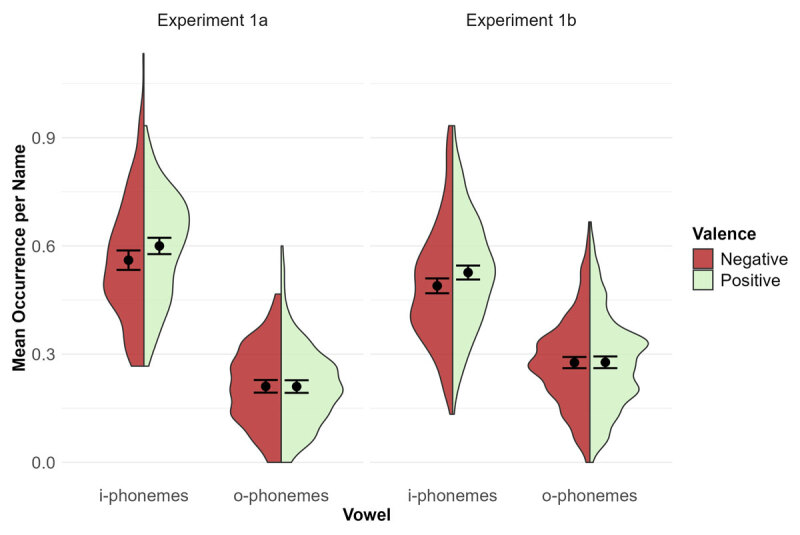
Main Analysis Experiment 1. *Note*. Mean occurrence of i-phonemes and o-phonemes per name depending on valence of the face to which participants had to give a name in Experiment 1a (left panel) and Experiment 1b (right panel). The dots with error bars represent means with 95% confidence intervals. The shapes are density plots, where greater width indicates higher frequency of occurrence.

i-phonemes occurred more frequently in names for positive (*M* = 0.60, *SE* = 0.01) than negative faces (*M* = 0.56, *SE* = 0.01), *t*(155) = 2.10, *p* = .037, *d*_z_ = 0.17, 95% CI [0.01, 0.35]. However, for o-phonemes there was no significant difference for negative (*M* = 0.21, *SE* = 0.01) compared to positive faces (*M* = 0.21, *SE* = 0.01), *t*(155) = 0.05, *p* = .961, *d*_z_ = 0.00, 95% CI [–0.15, 0.18]. This difference was hypothesized to result in a significant interaction of valence and vowel type. However, the predicted interaction did not reach significance, *F*(1, 155) = 3.23, *p* = .074, \[
\eta_{\mathrm{p}}^2 = .020
\] , 90% CI [.000, .071]. There was no main effect of valence, *F*(1, 155) = 2.94, *p* = .088, \[
\eta_{\mathrm{p}}^2 = .019
\] , 90% CI [.000, .068]. The main effect of vowel type was significant, *F*(1, 155) = 1202.99, *p* < .001, \[
\eta_{\mathrm{p}}^2 = .886
\] , 90% CI [.861, .904]; i-phonemes (*M* = 0.58, *SE* = 0.01) occurred more frequently than o-phonemes (*M* = 0.21, *SE* = 0.01), see Figure 2.

In addition to the pre-registered analyses, we also explored alternative analyses strategies. Coding i-phonemes and o-phonemes as binary (i.e., present vs. absent), yields similar though stronger effects and a significant interaction. (Logistic) linear mixed model analyses for this as well as the following experiment yield modeling issues, even when removing random effects, presumably because participants were only asked to provide one name per stimulus. For these as well as exploratory analyses on other rounded vowels for all experiments, see https://osf.io/925ky/.

#### Experiment 1b

As in Experiment 1a, i-phonemes occurred more frequently in names for positive (*M* = 0.53, *SE* = 0.01) than negative faces (*M* = 0.49, *SE* = 0.01), *t*(242) = 2.48, *p* = .014, *d*_z_ = 0.16, 95% CI [0.03, 0.29]. However, o-phoneme occurrences again did not differ significantly for negative (*M* = 0.28, *SE* = 0.01) and positive faces (*M* = 0.28, *SE* = 0.01), *t*(242) = 0.08, *p* = .939, *d*_z_ = 0.00, 95% CI [–0.14, 0.12]. The interaction again did not reach significance, *F*(1, 242) = 3.32, *p* = .070, \[
\eta_{\mathrm{p}}^2 = .014
\] , 90% CI [.000, .047]. The main effect of valence was significant, *F*(1, 242) = 5.21, *p* = .023, \[
\eta_{\mathrm{p}}^2 = .021
\] , 90% CI [.002, .060], with more target vowels in names for positive faces (*M* = 0.40, *SE* = 0.01) than in names for negative faces (*M* = 0.38, *SE* = 0.01).The main effect of vowel type was also significant, *F*(1, 242) = 583.95, *p* < .001, \[
\eta_{\mathrm{p}}^2 = .707
\] , 90% CI [.661, .745], with more occurrences of i-phonemes (*M* = 0.51, *SE* = 0.01) than o-phonemes (*M* = 0.28, *SE* = 0.01), see Figure 2.

#### Joint Analysis Experiments 1a and 1b

The pre-registered power analyses for Experiments 1a and 1b were based on simple comparisons. For the interactions, additional simulation-based power-analyses (Lakens & Caldwell, 2021) using the respective sample sizes and assumed effect sizes (*d*_z_ = 0.20 and *d*_z_ = 0.16) for both simple effects yielded 95% power in both Experiments. However, as o-occurrences did not vary with valence, the effect size for the interaction was much smaller than expected and did not reach significance. A simulation-based power-analysis using *d*_z_ = 0.20 for the valence effect for i-phonemes and *d*_z_ = 0.00 for the valence effect for o-phonemes yields a required sample size of 395 to achieve 80% power for the interaction. Thus, to explore this interaction with increased power, we conducted a joint analysis, including all 399 participants. In this analysis, the interaction between valence and vowel type was significant, *F*(1, 398) = 6.42, *p* = .012, \[
\eta_{\mathrm{p}}^2 = .016
\] , 90% CI [.002, .042].

## Study 2: Corpus Analysis

To examine whether the association between positive valence and i-occurrence in names generalizes beyond faces, we analyzed a corpus of names described in Nett et al. (2020). This corpus consists of 2000 representative unique German first names that occur both in a newspaper corpus and name dictionary and were selected to be sufficiently distinct. For each name, the corpus contains participant ratings on several person perception dimensions. We examined whether i-phoneme occurrence and o-phoneme occurrence predict likability ratings.

### Method

Name evaluations were assessed by Nett et al. (2020) by asking participants to rate a typical person with a given name on several 7-point scales. Participants evaluated, for example, how attractive, likable, religious, and independent a typical person with a specific name is, with higher values indicating higher prevalence of the evaluated property. Consistent with Experiment 1b, we concentrated on likability ratings.^3^ Each name in the corpus by Nett et al. (2020) was phonologically coded as in Experiment 1.

### Results

Names were the unit of analysis, resulting in a between-item analysis. As likability ratings were higher for female (*M* = 4.40, *SE* = 0.01) than male names (*M* = 4.23, *SE* = 0.01), *t*(1993) = 11.15, *p* < .001, *d* = 0.50, 95% CI [0.41; 0.59], and i-phonemes occurred more frequently in female (61.4%) than male names (46.7%), χ^2^(1) = 43.17, *p* < .001, name gender is a potential confound (see also Cutler et al., 1990; Whissell, 2006). Therefore, we added name gender as a factor to all analyses.

**Figure 3:**
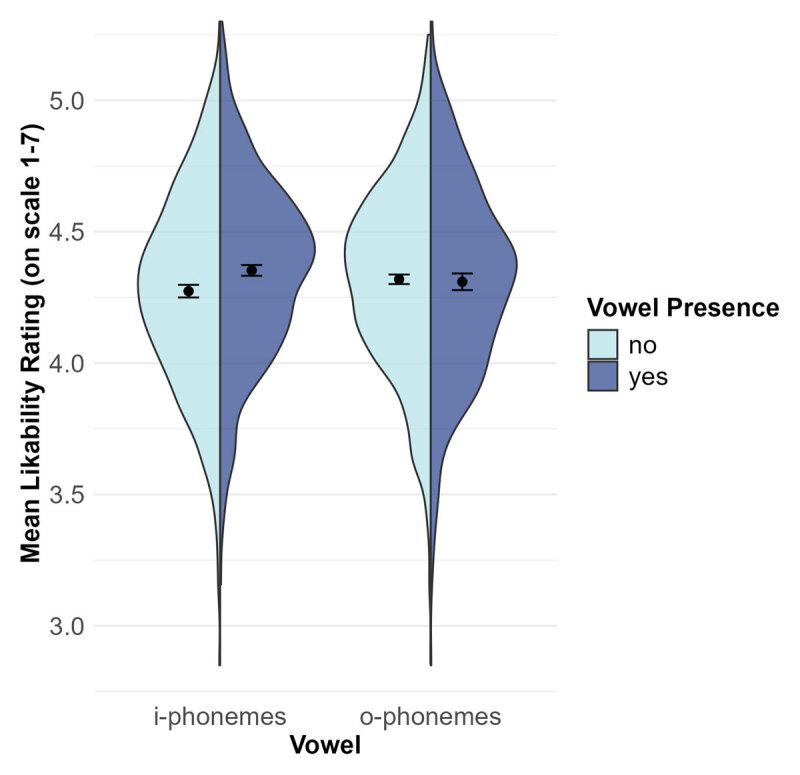
Main Analysis Study 2. *Note*. Mean likability rating of a typical name bearer depending on the occurrence of i-phonemes and o-phonemes in their name in the corpus provided by Nett et al. (2020). The dots with error bars represent means with 95% confidence intervals and the shapes are density plots.

To examine whether likability is predicted by the occurrence of i-phonemes and o-phonemes, name gender (male vs. female) and dichotomized predictors whether the name contained i-phonemes, o-phonemes as well as their interaction were entered into an ANOVA.^4^ Likability was predicted by both, gender, *F*(1,1996) = 125.15, *p* < .001, \[
\eta_{\mathrm{p}}^2 = .059
\] , 90% CI [.043, .076] (see above), and i-occurrence *F*(1,1996) = 12.81, *p* < .001, \[
\eta_{\mathrm{p}}^2 = .006
\] , 90% CI [.002, .013]. People with at least one i-phoneme in their name were judged to be more likable (*M* = 4.35, *SE* = 0.01) than people with no i-phoneme in their name (*M* = 4.27, *SE* = 0.01). The main effect for o-occurrence was not significant, *F*(1,1996) = 1.16, *p* = .282, \[
\eta _{\mathrm{p}}^2 > {\mathrm{.001}}
\] , 90% CI [.000, .004], with similar likability ratings for people whose name does contain o-phonemes (*M* = 4.31, *SE* = 0.02) and does not contain o-phonemes (*M* = 4.32, *SE* = 0.01). The interaction was also not significant, *F*(1,1996) = 0.81, *p* = .369, \[
\eta _{\mathrm{p}}^2 > {\mathrm{.001}}
\] , 90% CI [.000, .003], see Figure 3. Reversing the analysis, that is, predicting vowel occurrences from likability ratings, yielded qualitatively identical results. Thus, as hypothesized, i-phonemes are associated with increased likability ratings, that is, with positive valence. However, again, o-phonemes are not significantly associated with negative valence.

## General Discussion

Valence is associated with speech sounds. Prominently, /i/ has been found to be associated with positive valence and /o/ with negative valence (Körner & Rummer, 2022; Rummer et al., 2014; Rummer & Schweppe, 2019). The present research tested these associations using a new experimental paradigm where participants are asked to choose any name from memory they feel suitable for a given face, thereby examining whether previously observed sound-symbolic associations generalize to real first name in an unrestricted paradigm.

In two experiments and an analysis of a representative name corpus, we found that i-phonemes are associated with positive valence. Specifically, consistent with previous findings, faces with positive (vs. negative) emotional expressions (Experiment 1a) and faces high (vs. low) in likability (Experiment 1b) were given names that contained more i-phonemes. Similarly, in the name corpus, likability of a name was predicted by i-occurrence (Study 2). However, in contrast to our hypothesis, there was no significant association between o-phonemes and negative valence; o-occurrence neither differed depending on face valence (Experiments 1a & 1b) nor did it predict likability judgments (Study 2).

Notably, these results of Experiments 1a and 1b are quite similar, indicating that associations between i-phonemes and positive valence do not depend on the subtlety of the valence manipulation (which was greater in Experiment 1b than Experiment 1a) or the nature of the valence manipulation (likability in Experiment 1b and facial expressions in Experiment 1a) or the artificiality of the stimuli (computer generated in Experiment 1b and photographs in Experiment 1a). In other words, we find that positive faces are associated with i-phonemes in names, both when valence is manipulated via facial features even though all faces depict neutral expressions (Experiment 1b; see e.g., Oosterhof & Todorov, 2008) and when valence is manipulated via facial expression even though facial features are identical (Experiment 1a). Therefore, aspects of neither facial features nor facial expressions can completely account for the present findings, making valence differences the most parsimonious explanation for the present findings.

A hitherto open question concerns generalizability across cultures. There is some evidence that the two valence manipulations, facial expression and likability, generalize across cultures (e.g., Elfenbein & Ambady, 2002; Jones et al., 2021). Moreover, previous research indicates some cross-cultural generalizability of vowel–valence associations (e.g., Körner & Rummer, 2023; Yu et al., 2021). Whether this is also the case for real first names needs to be tested by future research.

A feature that the naming paradigm shares with corpus studies and similar research (e.g., Westbury et al., 2018) consists in having to choose word form features to examine. The sheer number of possibilities (graphemes, phonemes, phonetic features, biphones, bigrams, or any of these for specific word positions, e.g., nasality of the first phoneme), necessitates selection.^5^ A frequently employed analysis strategy concentrates on phonetic features, such as voicing. This greatly reduces the number of analyses but entails the risk of choosing features that mask instead of reveal sound symbolic associations (Monaghan & Fletcher, 2019). For example, when examining valence–vowel associations, De Zubincaray et al. (2023) included vowel height while Sidhu et al. (2022) included vowel frontness. However, /y/, a high front vowel, has been found to be associated with negative valence while /i/, another high front vowel, is associated with positive valence, indicating that neither height nor frontness drives vowel–valence associations (Körner & Rummer, 2022). Thus, sub-optimal feature choice is a possible explanation why some research found no evidence for vowel–valence associations. Similarly, for consonants, the choice of phonetic features and the individual consonants used for these features might be responsible for the inconsistent results. Relatedly, Adelman et al. (2018) argue that phonemes instead of phonetic features drive valence sound symbolism.

In contrast to the vast majority of previous studies, which use consonant–vowel–consonant–vowel pseudo-words, the present experimental paradigm does not pose any restrictions on word structure, so that complex syllables and their combinations are possible. This complexity invites more sophisticated analyses strategies than typically performed. Hitherto, research has concentrated on associations between meaning and frequency of phonetic features or individual phonemes, with some research additionally including predictors for first or last phoneme (e.g., Adelman et al., 2018; Westbury et al., 2018). However, phoneme combinations or whole syllables could more adequately represent co-articulation and accordingly predict sound symbolism better than individual phonemes. Whether this is indeed the case, which kind of phoneme combinations should be examined, and how this can be done while still maintaining a manageable number of predictors needs to be examined by future research.

Here, we report only analyses on overall vowel frequencies for which we had a theory-based hypothesis. This analysis enables a comparison to previous research in valence sound symbolism (e.g., Körner & Rummer, 2023; Rummer & Schweppe, 2019), enabling conclusions whether previous findings replicate when using the naming paradigm. For exploratory analyses on consonant features, see https://osf.io/b469r. The present open data enable re-analyses with more sophisticated methods that incorporate the interactivity of phonetic components, which could foster a more nuanced understanding of complex word form–meaning associations.

A disadvantage of the present approach, shared by any paradigm that uses real words, is its sensitivity to baseline frequency of word features. In line with vowel occurrence in first names, we always observed a main effect of vowel, with i-phonemes being more frequent than o-phonemes. This main effect was unproblematic in the current research, as we hypothesized occurrence differences depending on an additional factor, valence. Accordingly, we suggest using the naming paradigm only to examine the influence of word-external manipulations on word form feature occurrence. Moreover, very infrequent target word form features might have low statistical power. Thus, when examining features that are very seldom in typical names, pseudo-word paradigms could be better suited than the naming paradigm.

Compared to previous experiments that employed similar stimuli to examine vowel–valence associations but used pseudo-names, the present effect sizes are notably smaller. The association of i-phonemes and positive valence reached *d*_z_ = 0.15–0.20 compared to *d*_z_ = 0.40–0.80 in most other studies (e.g., Körner & Rummer, 2023; Rummer & Schweppe, 2019). This is probably caused by additional variance resulting from semantic associations with real names. For example, names might have been selected because a stereotype for a name matched a stereotype a participant had about a face. Accordingly, the most frequent names for positive faces were also very frequent for negative faces and vice versa (see https://osf.io/2v8gm). For the present analyses, associations like these increase error variance, which reduces effect sizes (for additional reasons why sound symbolism in real words is expected to yield smaller effect sizes than sound symbolism in pseudo-words, see Sidhu & Pexman, 2019). In the present study, we wanted to test whether valence–vowel associations are robust enough to show in spite of these sources of error variance.

The association between o-phonemes and negative valence, which had been smaller than the effects for i-phonemes in previous studies, was not significant in the present research. Thus, the association between o-phonemes and negative valence could be less stable than the association between i-phonemes and positive valence and not generalize to real names; alternatively, the association between o-phonemes and negative valence could be too small to be detected in the present research. A reason why this association could be smaller and less stable is its indirect nature. According to the articulatory explanation of valence sound symbolism (see Introduction), i-phonemes are associated with positive valence because articulating i-phonemes involves muscles that are also involved in smiling, which are directly associated with positive affect. In contrast, o-phonemes are postulated to be associated with negative valence because their articulation involves muscles that inhibit smiling, constituting only an indirect association with negative valence. The indirectness of the postulated association between o-phonemes and negative valence could explain its smallness and its fragility.^6^

Sound symbolism has been argued to be a key component of language evolution (e.g., Johansson et al., 2020; Vigliocco et al., 2014). A basic function of language is referentiality, mapping a linguistic sign (e.g., a word) to a referent, because referentiality is necessary for communicating about something not present in the here and now (Imai & Kita, 2014; Perniss & Vigliocco, 2014). By asking participants to provide suitable names, the naming paradigm taps into this function of language. In other words, the naming paradigm naturally assesses vital psychological aspects of referentiality, a key function of language. Thereby, the naming paradigm is ideally suited to further our understanding of the role of sound symbolism in diachronic language development.

## Data Accessibility Statement

Data, analysis scripts, materials, and links to the pre-registrations can be found at https://osf.io/925ky/.

## Footnotes

Other problems have also been discussed, for example, poor inter-rater agreement concerning semantic meaning (see de Zubicaray et al. 2023).

This can be remedied by using pseudo-word experiments to test results from corpus analyses, see Schmidtke and Conrad (2024).

The results are similar for all warmth-related evaluations and attractiveness, whereas results for other dimensions differ. For details, see https://osf.io/925ky/?view_only=f6509c789ce14699bc6e16e46f6fe4d6.

Using the number of i-phonemes and o-phonemes instead leads to qualitatively identical results, see https://osf.io/925ky/?view_only=f6509c789ce14699bc6e16e46f6fe4d6.

Additionally, structural word features, such as word length (Lewis & Frank, 2016) or syllable structure (Maschmann et al., 2024) have also been found to be associated with word meaning and could therefore also be included.

Notably, an alternative account of vowel–valence associations makes symmetric predictions, that is, predictions that /i/ is associated with affiliative signals and /o/ is associated with threatening signals. Specifically, Ohala (1984, 1996) postulated that an o-face (smiling) increases (decreases) the vocal tract length, leading to lower frequencies of utterances, signaling threat (affiliation).
